# Designing adhesive hydrogels for oral diseases treatment

**DOI:** 10.1016/j.mtbio.2026.102911

**Published:** 2026-02-12

**Authors:** Yufei Peng, Zhisheng Jiang, Shusen Xu, Liming He, Tong Jiang, Yujie Yang, Xiaoyan Xie, Lanjie Lei

**Affiliations:** a Department of Stomatology, Changsha Stomatological Hospital, Changsha, Hunan, 410004, China; b School of Stomatology, Hunan University of Chinese Medicine, Changsha, Hunan, 410208, China; c Department of Stomatology, The Second Xiangya Hospital of Central South University, Changsha, Hunan, 410011, China; d Key Laboratory of Artificial Organs and Computational Medicine in Zhejiang Province, Shulan International Medical College, Institute of Translational Medicine, Zhejiang Shuren University, Hangzhou, Zhejiang, 310015, China

**Keywords:** Adhesive hydrogel, Oral disease treatment, Bioadhesion, Drug delivery

## Abstract

Localized drug delivery in the oral cavity is challenging owing to its wet, dynamic, and microbiologically complex nature. Adhesive hydrogels have attracted increasing attention for their ability to adhere under wet conditions, provide sustained drug release, and respond to pathological environments. This review provides a structured perspective by categorizing advancements into a three-level hierarchy encompassing molecular bonding, network reinforcement, and system-level adaptation. Chemical motifs for interfacial stability and responsive designs are systematically evaluated to enhance site-specific compatibility. Unlike previous reports that primarily cataloged material types, this study distinguishes itself through a rigorous quantitative comparison between experimental hydrogels and clinical gold standards across diverse oral pathologies. A primary differentiator is the emphasis on biomechanical crosstalk where microbial enzymatic activity and mechanical fatigue concurrently dictate the longevity of the adhesive interface. Furthermore, the work identifies systemic disconnects in current testing methodologies and advocates for integrated evaluation paradigms that simulate simultaneous physical and biological stressors. By synthesizing these granular insights, this review offers a comprehensive roadmap for achieving stable and efficient therapy in next-generation oral medicine.

## Graphical abstract

**Figure:**
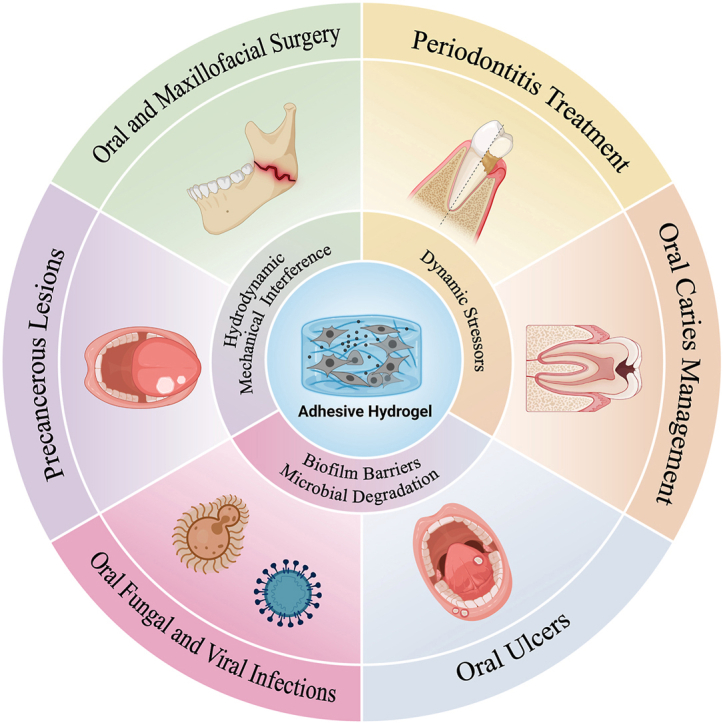

## 1 Introduction

Oral diseases are highly prevalent chronic noncommunicable conditions that impose a significant health and economic burden on communities worldwide [1,2]. According to the Global Burden of Disease Study, over 3.5 billion people suffer from conditions, such as dental caries, periodontal disease, oral ulcers, oral cancer, and precancerous lesions, with prevalence rates increasing each year [[3], [4], [5]]. Oral diseases not only cause local tissue destruction, severely impairing masticatory function and nutritional intake, but also synergistically exacerbate their impact on systemic diseases through chronic inflammatory pathways. These factors play a crucial role in the development of various chronic conditions, such as hypertension, diabetes, and cardiovascular ailments [[6], [7], [8], [9]]. This local–systemic interaction continually intensifies the public health burden and exacerbates the strain on primary healthcare systems in terms of manpower and medication, a situation that is particularly acute in low- and middle-income countries [10].

Effective management of oral diseases often depends on localized therapy; however, the oral cavity presents a highly dynamic and bioactive environment for drug delivery. Owing to high salivary flow, mechanical stress from mastication and swallowing, and a complex microbial ecology, the oral mucosa significantly reduces the residence time of conventional formulations [11]. Most oral disease lesions are associated with a sophisticated inflammatory microenvironment characterized by reduced pH levels, accumulation of reactive oxygen species (ROS), and heightened protease activity [[12], [13], [14]]. Conventional formulations, such as pastes, patches, and mouthwashes, frequently fail to maintain prolonged adhesion at the target site owing to salivary washout, physical abrasion, and enzymatic degradation [[15], [16], [17], [18]]. Consequently, these systems suffer from short retention times, sub-therapeutic drug concentrations, and poor patient compliance, ultimately limiting their clinical efficacy.

In recent years, hydrogels have emerged as promising candidates for localized drug delivery, owing to their high water content, biocompatibility, and tunable physical properties [[19], [20], [21]]. However, conventional hydrogels often lack the ability to adhere firmly to wet and dynamic oral tissues, leading to premature displacement and reduced therapeutic efficacy. Adhesive hydrogels represent an advanced category of biomaterials designed to overcome these limitations by providing robust interfacial adhesion, typically achieved through bioinspired chemical motifs, physical interactions, and microstructural adaptation. These materials, with their robust wet adhesion and programmable responsiveness to environmental stimuli, pave the way for intelligent, sustained drug release precisely tailored to the pathological microenvironment [[22], [23], [24], [25]].

**Fig. 1:**
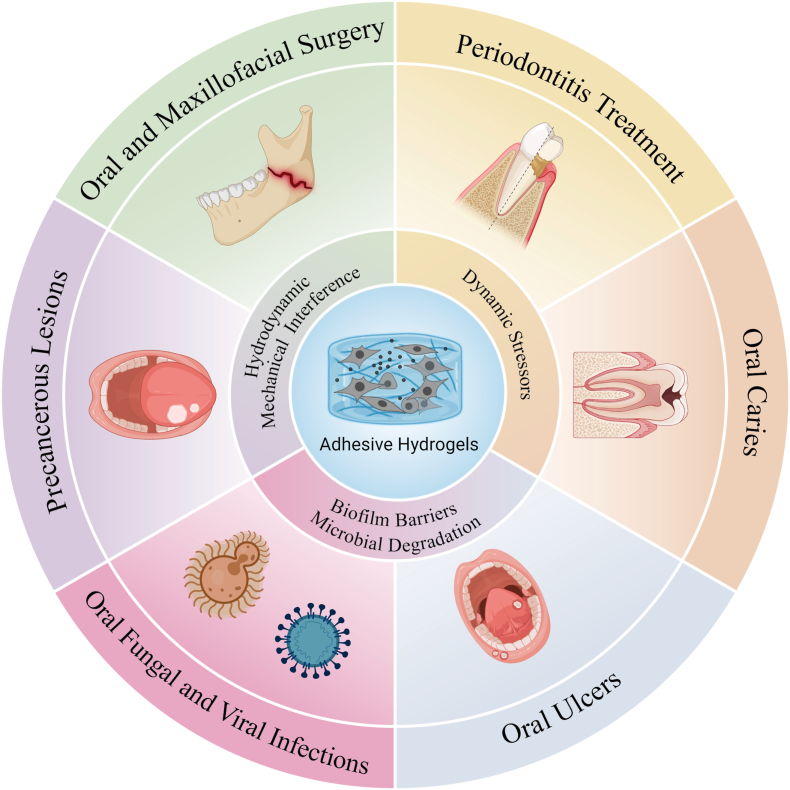
Adhesive hydrogels for localized treatment of oral diseases.

This review systematically examines the development of adhesive hydrogels for localized oral therapy through an integrated analysis of adhesion mechanisms and multi-scale design strategies. This study aims to provide a systematic and forward-looking analysis that offers several unique advantages over existing reports. Beyond merely cataloging materials, this study establishes a multi-scale design framework spanning the molecular, network, and system levels to explicitly link fundamental chemical motifs with functional performance. Furthermore, this study distinguishes itself by critically evaluating therapeutic applications through the lens of clinical translation barriers and explicitly addressing the quantitative mismatch between laboratory designs and site-specific physiological challenges. By synthesizing the most recent breakthroughs in intelligent responsiveness and cross-hierarchical strategies from the past three years, this study offers a timely and granular perspective on the future trajectory of the field. Fig. 1 presents a conceptual overview, positioning adhesive hydrogels as a versatile platform at the interface between the complex oral environment and major disease types and highlighting their potential for targeted intervention across a range of oral pathologies.

## 2 Oral Mucosal Barriers and drug delivery strategies

**Fig. 2:**
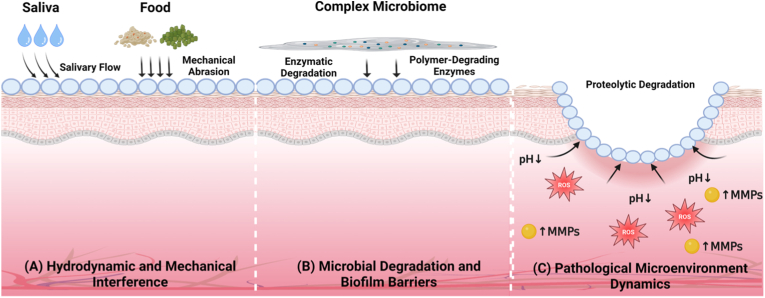
Oral Mucosal Barriers. (A) Hydrodynamic and Mechanical Interference: Salivary flow and food contact represent the primary sources of physical shear and abrasion that challenge material retention. (B) Microbial Degradation and Biofilm Barriers: The complex microbiome forms biofilms that produce polymer-degrading enzymes, leading to material enzymatic degradation. (C) Dynamic Stress Interference from the Inflammatory Microenvironment: Pathological conditions are characterized by elevated proteolytic activity, which accelerates the breakdown of hydrogel matrices and drug carriers.

The unique anatomical structure and microenvironment of the oral mucosa serve as fundamental biological barriers, posing significant challenges for drug delivery. Consequently, the design of targeted delivery systems must prioritize these barriers [26]. Compared to other tissues, the oral cavity presents a particularly demanding environment for localized therapy. As summarized in Fig. 2, this challenging environment encompasses hydrodynamic and mechanical interference (Fig. 2A) resulting from salivary flow and mastication, microbial degradation and biofilm barriers (Fig. 2B) formed by the complex oral microecology, and dynamic stress interference (Fig. 2C) originating from the pathological inflammatory microenvironment. Within this dynamic setting, conventional drug formulations are easily washed away, diluted, or degraded, which impedes the maintenance of effective concentrations and sustained therapeutic action. Therefore, achieving stable adhesion, overcoming these multiple barriers, and ensuring controlled drug release are the primary challenges that must be overcome for advancements in localized treatment strategies in oral healthcare.

### 2.1 Hydrodynamic and mechanical interference

The oral mucosa consists of epithelial cells that continuously secrete a sophisticated mucous surface gel network, forming a mucus layer [27]. Although this maintains a moist environment, the hydrodynamic properties significantly challenge local drug delivery systems. Constant saliva secretion, tongue movements, swallowing, and the introduction of exogenous food create a highly dynamic and mechanically complex setting with elevated shear forces. For delivery systems such as patches or hydrogels, once initial adhesion is established, materials often experience detachment, slippage, or structural failure due to shear stress, leading to rapid drug loss and termination of therapeutic effects [[28], [29], [30]].

Quantitative comparative studies have demonstrated that commercial saliva substitutes based on cellulose or gums exhibit extremely low mucoadhesion of typically less than 1 kPa, which results in brief wet residence times and insufficient lubrication. In contrast, Pec-PG hydrogels, which incorporate polyphenolic functional groups like pyrogallol (PG) to enable self-crosslinking via autoxidation, achieve a maximum adhesion force of approximately 6–7 kPa on ex vivo porcine tongue tissue [31]. This quantitative enhancement makes Pec-PG hydrogels significantly superior to poorly adhesive commercial products and enables them to better withstand hydrodynamic interference within the oral cavity. Consequently, high-performance hydrogels must achieve an interfacial adhesion strength of at least 6 kPa through a strategic network design to ensure stable and long-term retention under complex hydrodynamic conditions.

### 2.2 Microbial degradation and biofilm barriers

The oral cavity, as an open environment, harbors a diverse and stable microbial community that includes bacteria, fungi, and viruses, forming a complex adhesive biofilm ecosystem. Oral microecology harbors degradative enzymes and microbial metabolites that can compromise the structural integrity of hydrogels. This process shortens the drug release cycle and may result in premature failure of the delivery system [[32], [33], [34]]. The capacity of a hydrogel to resist such biological erosion is directly linked to its internal cohesive strength and polymer charge. Quantitative comparative studies have revealed divergent dissolution kinetics in artificial saliva, establishing a clear hierarchy of material stability. For instance, hydroxyethyl cellulose (HEC) loses nearly 100% of its mass within 30 min, whereas xanthan gum (XTGM) and carboxymethyl cellulose (CMC) maintain a superior structural cohesion. This results in a quantitative residence time hierarchy of CP < HEC < HA < ALG < PCP < CMC < XTGM [35]. Accordingly, designing delivery systems to overcome microbial barriers necessitates the optimization of cohesive networks to prolong the residence time. Furthermore, to effectively bypass biofilm barriers with thicknesses of 50–100 μm, hydrogels must be engineered to facilitate drug penetration depths exceeding 50 μm to ensure therapeutic efficacy against deep-seated pathogens [36].

### 2.3 Dynamic stress interference from the inflammatory microenvironment

The dual barrier system of the oral mucosa, consisting of a stratified squamous epithelium and a network of immune sentinel cells, undergoes dynamic remodeling during inflammatory states [[37], [38], [39]]. Oral diseases, such as periodontitis and mucositis, are frequently accompanied by chronic inflammatory responses. In such cases, the microenvironment is characterized by stress conditions, such as dysregulated ROS levels, activated pro-inflammatory signaling, and localized pH drops ranging from 5.0 to 6.5 [40]. The immune barrier is continuously exposed to inflammatory signals, including abundant oral microbiota and their metabolic products, as well as antigens from food and air [[41], [42], [43]]. The abnormal upregulation of extracellular matrix-degrading enzymes, particularly MMP-2 and MMP-9, significantly accelerates the structural degradation of hydrogel materials. Quantitative evidence has indicated that MMP-9 exhibits potent proteolytic activity, capable of causing over 95% mass loss in hydrogel networks within 14 days, whereas non-enzymatic hydrolysis contributes an additional 2–6% degradation load. These factors not only hasten the failure of the hydrogel carrier and disrupt drug delivery but also cause interfacial detachment, which severely compromises therapeutic precision [44,45]. Consequently, achieving a quantitative balance between biostability and on-demand degradation to withstand this intense proteolytic stress is essential for ensuring the durability of localized oral therapies.

## 3 Definition of adhesive hydrogels

Adhesive hydrogels are a class of functional soft materials featuring a three-dimensional network structure [[46], [47], [48], [49]] and interfacial adhesion capabilities. They can achieve stable adhesion on wet bioactive surfaces and are widely used in various medical applications, including localized drug release, tissue fixation, and hemostatic sealing [[50], [51], [52], [53], [54]]. These materials are typically constructed from highly hydrophilic polymers through physical or chemical crosslinking, which gives them excellent biocompatibility and tunable mechanical properties. Consequently, they have emerged as a prominent frontier in biomedical materials research.

Studies have incorporated biomimetic adhesive units, intelligently responsive modules, and microstructural designs into traditional hydrogels to enhance their performance at biological interfaces, particularly in hydrated, dynamic, and protein-rich environments. This incorporation enables instantaneous wet adhesion to hydrated tissue surfaces, resulting in a durable interface that withstands dynamic physiological stresses. Early strategies focused on mimicking the chemical mechanisms of strongly adherent organisms. For instance, mussel-inspired hydrogels incorporate catechol groups into polymers, enabling hydrogels to establish strong and durable adhesions to various biological and nonbiological surfaces in aqueous environments [55,56]. Acknowledging that chemical groups alone are insufficient under complex stresses, research has shifted toward optimizing interfacial energy and designing micro- and nanoscale structures. Zhao et al. developed a hydrogel construction strategy focused on regulating interfacial energy, which endowed the hydrogel with exceptionally high shear and tensile strengths while enabling functional and stable connections across various material surfaces [57]. Simultaneously, current research trends aim to imbue hydrogels with environmental responsiveness and self-adaptability. Adhesive hydrogels utilizing supramolecular strategies [58,59] and stimulus responsiveness, including dynamic covalent bonds and non-covalent supramolecular interactions, have produced intelligent hydrogel systems capable of reversible, stimulus-responsive adhesion, self-healing, and repeatable closure [60,61].

These studies have shown that optimizing molecular interactions within hydrogels significantly enhances their interfacial adhesion in complex biological environments. Advances in interfacial materials science have transformed adhesive hydrogels from traditional passive adhesion systems into intelligent interfacial materials with synergistic multimechanical interactions and environmental adaptability.

## 4 Construction strategies for adhesive hydrogels: multi-scale design and functional integration

The successful application of adhesive hydrogels stems from a comprehensive engineering design that spans molecular to macroscopic levels and static to dynamic conditions. The fabrication of these hydrogels is not a mere combination of isolated techniques but represents an integrated system of multi-scale synergistic actions [62,63]. The efficacy of an adhesive system depends on the hierarchical synergy among the molecular, network, and system design levels. Molecular interactions establish the fundamental interfacial contact associated with adhesion work, whereas network architectures dissipate mechanical stress to prevent bulk failure through enhanced fracture toughness. Simultaneously, system-level integration enables active adaptation to the dynamic oral environment. Sustainable interfacial stability is achieved only through the coordinated alignment of these dimensions, ensuring that molecular bonds are reinforced by a resilient network and optimized by an intelligent system. As summarized in Table 1, the design paradigms can be systematically categorized based on their design level, providing a framework for understanding the current landscape of the field.

**Table 1:** Multiscale design, advantages, and challenges of adhesive hydrogels for oral therapeutics.

Design Level	Key Strategies & Representative Systems	Strengths and Limitations
**Molecular-Level**	- **Strategies**: Bioinspired motifs (e.g., catechol), dynamic covalent chemistry, molecular sutures (e.g., PAADA/PEI). - **Systems**: Catechol-functionalized hydrogels, PBA-based hydrogels.	- **Advantages**: Provides fundamental wet adhesion, versatile responsiveness, enables topological entanglement for enhanced interfacial toughness. - **Challenges**: Balancing robust adhesion with long-term biocompatibility; dependency on network integration for mechanical dissipation.
**Network-Level**	- **Strategies**: Enhanced robustness via DN/IPN structures, controlled crosslinking, emerging dry-state/crystal refinement strategies for cross-scale programming. - **Systems**: PAM-Alginate DN hydrogels, anti-swelling (AS) hydrogels.	- **Advantages**: Achieves high toughness, maintains integrity under stress, protects molecular-level bonds from mechanical and swelling-induced failure. - **Challenges**: Evaluation often static; need to simulate concurrent oral stressors (saliva, biofilm) for predictive validation.
**System-Level**	- **Strategies**: Smart adaptation via Janus structures & biomimetic microstructures, intelligent responses (pH, enzyme, light), scalable manufacturing (e.g., UV-photolithography). - **Systems**: Convertible Janus patches, MMP/pH-sensitive hydrogels, 3D shape-changing architectures.	- **Advantages**: Enables on-demand adhesion & targeted drug release, actively coordinates molecular and network functions for site-specific adaptation. - **Challenges**: High complexity poses hurdles for cost-effective, large-scale manufacturing and regulatory approval.
**Performance Validation**	- **Strategies**: Critical analysis of compartmentalized tests, advocacy for integrated multi-factor bioreactors. - **Systems**: Dynamic flow chambers, simulated mastication apparatus, emerging integrated testing platforms.	- **Advantages**: Generates data under simulated conditions; integrated approaches essential to capture bio-mechanical crosstalk and predict clinical durability. - **Challenges:** Lack of standardized protocols that simultaneously replicate mechanical fatigue, salivary flow, and active biofilm challenges.

### 4.1 Molecular-level design: Building the chemical foundation for adhesion

**Fig. 3:**
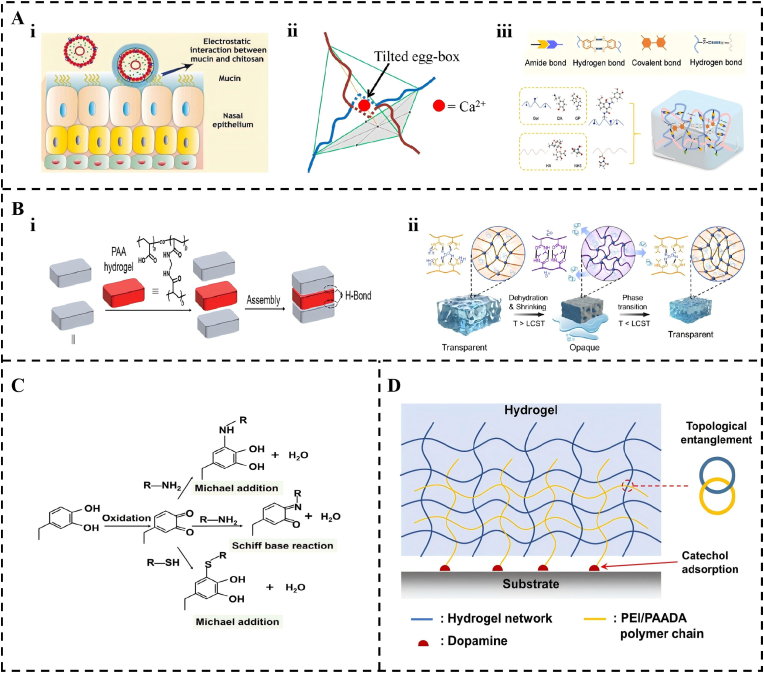
The chemical basis of adhesive interactions. (A) Adhesion of natural polymers. (i) Schematic diagram of chitosan electrostatic bridging. Reproduced with permission from Ref. [89]. Copyright 2024 Elsevier. (ii) Structure diagram of alginate "egg box". Reproduced with permission from Ref. [90]. Copyright 2013 American Chemical Society. (iii) Gelatin/HA Hydrated Adhesion Diagram. Reproduced with permission from Ref. [91]. Copyright 2022 Elsevier. (B) Functional design of synthetic polymers. (i) Assembly of PAA. Reproduced with permission from Ref. [92]. Copyright 2024 John Wiley and Sons. (ii) Mechanism illustration of the pure PNIPAM hydrogel and its LCST behavior. Reproduced with permission from Ref. [93]. Copyright 2024 American Chemical Society. (C) The catechol-mediated adhesion mechanism. Reproduced with permission from Ref. [94]. Copyright 2026 Elsevier. (D) Schematic depiction of the topological entanglement formed between PEI and PAADA polymers. Reproduced with permission from Ref. [95]. Copyright 2024 Elsevier.

At the molecular level, adhesion relies on both the selection of the polymer backbone and incorporation of functional adhesive groups. Natural polymers, such as chitosan [[64], [65], [66], [67], [68]], gelatin [[69], [70], [71], [72], [73]], hyaluronic acid (HA) [[74], [75], [76], [77], [78], [79]], and alginate (Alg) [[80], [81], [82], [83], [84]], inherently possess adhesive potential owing to their abundant amino, carboxyl, and hydroxyl groups [[85], [86], [87], [88]]. For instance (Fig. 3A), chitosan forms electrostatic bridges with negatively charged mucins [89,96,97], whereas alginate employs its G-units to create an egg-box structure via crosslinking with divalent cations, thereby adhering to tissues through physical encapsulation [90,[98], [99], [100]]. Gelatin and HA facilitate robust interfacial adhesion on hydrated tissue surfaces by establishing stable hydration layers through van der Waals forces and hydrogen bonding [91,101,102]. In contrast, synthetic polymers such as PAA [[103], [104], [105]] and PNIPAm [[106], [107], [108]] provide greater design flexibility and controllable properties (Fig. 3B) [109]. PAA achieves adaptive adhesion through carboxyl-mediated hydrogen bonding and pH-responsive assembly [92,[110], [111], [112]], whereas the lower critical solution temperature behavior of PNIPAm enables a temperature-triggered sol-gel transition, significantly enhancing its physical conformity with irregular mucosal topography [93,113,114].

The transition from physical adsorption to durable chemical bonding is achieved by integrating specific adhesive groups [115]. Inspired by mussel adhesive proteins, catechol chemistry is currently the most widespread and effective approach in wet adhesion research, with dopamine serving as a key molecular player [[116], [117], [118], [119]]. Catechol groups and their oxidized quinone forms can form dynamic covalent bonds with amine and thiol groups on tissue surfaces via Michael addition or Schiff base reactions in aqueous environments (Fig. 3C) [94,[120], [121], [122]]. This process operates synergistically with multiple non-covalent interactions, including hydrogen bonding, π–π stacking, and metal ion coordination [[123], [124], [125]], collectively enabling the formation of powerful and durable interfacial bonds. Beyond catechols, other dynamic systems like Schiff base formation and boronic ester bonds enable pH-sensitive or reversible binding [[126], [127], [128]].

Recent advancements have emphasized that the efficacy of these molecular bonds depends on their hierarchical coupling with the broader polymer network. Emerging molecular stitching techniques bridge the gap between molecular chemistry and network mechanics by utilizing biocompatible polymers as molecular sutures [129]. A representative example is the mussel-inspired polyelectrolyte system, which comprises catechol-functionalized polyacrylic acid (PAADA) and polyethyleneimine (PEI) (Fig. 3D) [95]. In this system, reversible dynamic physical interactions accelerate the diffusion of polymer chains into pre-existing hydrogel networks to achieve effective entanglement. This strategy synergistically combines direct catechol-mediated bonding with topological stitching, resulting in exceptional interfacial toughness, self-healing properties, and recyclability. Furthermore, the development of ultrasound-mediated cavitation over the past three years has introduced active strategies to drive these molecular sutures into low-permeability tissues, overcoming diffusion limits [130]. This multi-scale coordination ensures that the molecular-level foundation is structurally integrated into the macroscopic architecture, enabling it to withstand dynamic mechanical challenges.

### 4.2 Network-level construction: Regulating mechanical and stability performance

Building upon the molecular sutures established at the interface, network-level construction provides the mechanical framework necessary to protect these interfacial anchors from the dynamic oral environment [131]. The synergy between the molecular and network levels relies on hierarchical energy dissipation, wherein molecular interactions establish the initial attachment points, whereas the network architecture governs the overall work of fracture. A resilient network prevents premature delamination by transferring concentrated stress from molecular adhesive sites to the dissipative bulk through sacrificial bonds or sophisticated topological structures [132,133].

**Fig. 4:**
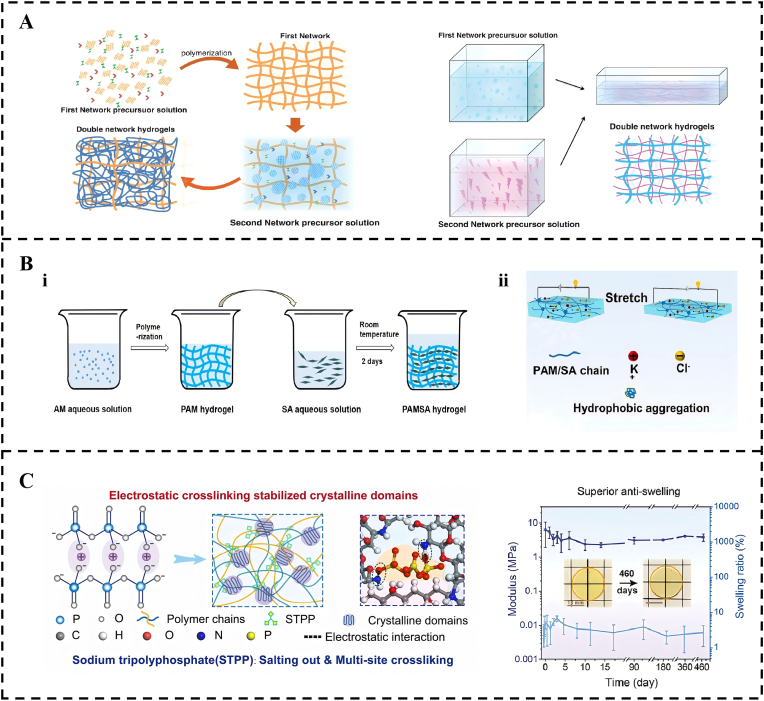
Network-Level Construction. (A) Schematic diagram illustrating the fabrication of double-network hydrogels through successive chemical crosslinking strategies, encompassing both the two-step polymerization and the molecular stent methods. Reproduced with permission from Ref. [138]. Copyright 2018 John Wiley and Sons. (B) PAMSA hydrogel. (i) PAMSA hydrogel production process. Reproduced with permission from Ref. [144]. Copyright 2021 Elsevier. (ii) Structure of the PAM/SA hydrogel. Reproduced with permission from Ref. [145]. Copyright 2024 Elsevier. (C) A novel design strategy for preparing AS-gel with superior anti-swelling property. Reproduced with permission from Ref. [146]. Copyright 2025 Elsevier.

The transition from molecular anchoring to network stability is exemplified by double-network [[134], [135], [136], [137], [138]] and interpenetrating network [[139], [140], [141], [142], [143]] architectures (Fig. 4A). A representative paradigm of this synergy is the use of bio-based micro-phase separation designs, such as non-isocyanate polyurethane embedded within entangled networks [147]. In these systems, the network functions as a dissipative reservoir where dynamic hydrogen bonds act as sacrificial units that balance stiffness and extensibility, ensuring that the interface remains intact even under extreme mechanical loads. Similarly, the polyacrylamide/sodium alginate hydrogel system integrates ionic sacrificial bonds with a covalent network to address the mechanical mismatch between materials and soft mucosal tissues (Fig. 4B) [144,145,148,149]. These network-level strategies provide the fundamental toughness required to withstand the cyclic stresses of mastication.

Over the past three years, emerging cross-hierarchical design technologies have further enhanced this synergy by manipulating the physical states of polymer networks. A prominent breakthrough is the dry-state cross-scale design, which utilizes controlled phase transitions to achieve superior mechanical properties. For instance, a crystal refinement-driven strategy employs a drying-induced assembly of layered structures, which, upon rehydration, forms a high-density hierarchical hydrogen-bond network. This approach achieves a remarkable toughness of 164.6 MJ per cubic meter by effectively dissipating energy while maintaining chain extensibility [150]. This represents a novel paradigm wherein macroscopic processing is used to program molecular-level ordering and bonding, thereby directly integrating fabrication, network structure, and function. Furthermore, leveraging the wet-to-dry transition facilitates volumetric shrinkage and enhances intermolecular interactions at solid interfaces, resulting in adhesion strengths exceeding 3 MPa [151]. These strategies demonstrate how macroscopic physical state transitions can drive molecular-level entanglement to achieve high-performance interfacial bonding.

Beyond mechanical reinforcement, maintaining stability within the moist oral cavity requires precise control over the swelling behavior [[152], [153], [154], [155]]. Conventional hydrogels often suffer from excessive swelling, which weakens molecular-level bonds and leads to delamination. To address this issue, recently proposed advanced anti-swelling strategies utilize high-density crosslinking or hydrophobic domains to generate internal compressive stresses that oppose the osmotic pressure of saliva (Fig. 4C) [146]. These network-level interventions, when combined with the rebuilding of phase continuity in systems such as SGG adhesives [156], protect the underlying chemical foundation from environmental degradation. By integrating these multi-scale mechanisms, the adhesive system remains functionally robust throughout the dynamic therapeutic period.

### 4.3 System-level integration: achieving interfacial adaptation and intelligent response

Building on the molecular-level chemical foundations and network-level mechanical stability, system-level integration shifts the action of adhesive hydrogels from passive attachment to active biological adaptation. This executive layer resolves the conflict between robust therapeutic adhesion and the need to minimize collateral friction using hierarchical synergistic strategies. Thus, system-level integration actively coordinates molecular functionalities and network properties to perform complex, spatiotemporal tasks at the biological interface.

**Fig. 5:**
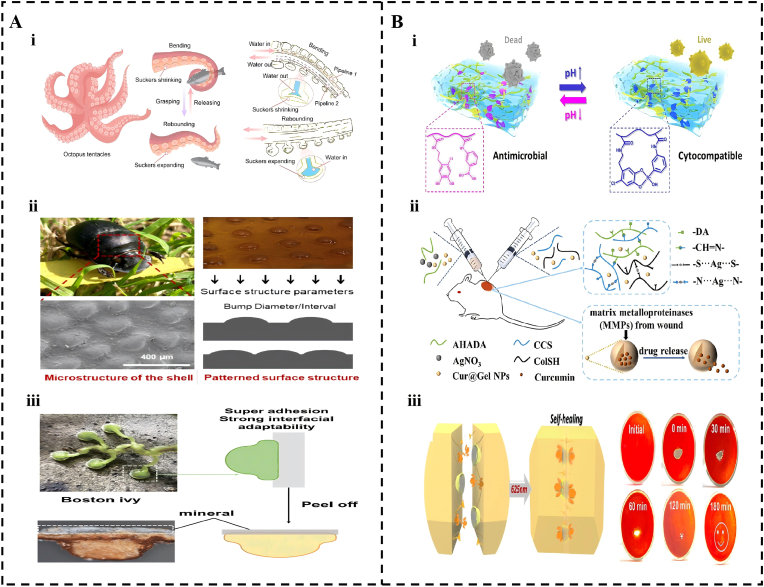
Biomimetic hydrogels and environment responsive hydrogel. (A) Biomimetic micro- and nanoscale structures enhancing physical adhesion. (i) Schematic of a biomimetic, hydraulically actuated hydrogel gripper; inspired by octopus suckers and arms for adaptive, gentle grasping of delicate underwater objects. Reproduced with permission from Ref. [166]. Copyright 2026 Springer Nature. (ii) Schematic of a dung beetle-inspired asymmetric adhesive hydrogel patch which designed with a patterned, non-adhesive top surface and a tissue-adhesive underside for effective internal trauma sealing and prevention of tissue adhesion. Reproduced with permission from Ref. [167]. Copyright 2025 American Chemical Society. (iii) Boston ivy-like branched tips for mechanical interlocking on rough surfaces. Reproduced with permission from Ref. [168]. Copyright 2025 Royal Society of Chemistry. (B) Environmentally responsive hydrogels enabling intelligent therapy. (i) PH responsive gel with enhanced adhesion and antimicrobial release in acidic pathological microenvironments. Reproduced with permission from Ref. [169]. Copyright 2022 Elsevier. (ii) MMP-sensitive hydrogel for inflammation-triggered, site-specific drug delivery. Reproduced with permission from Ref. [170]. Copyright 2023 American Chemical Society. (iii) Semiconvertible supramolecular gel allowing on-demand, atraumatic detachment. Reproduced with permission from Ref. [171]. Copyright 2022 American Chemical Society.

Geometric adaptation represents the first dimension of systemic design, focusing on spatial asymmetry and surface topography to meet specific oral clinical needs. Janus structures facilitate this by engineering an inner adhesive surface for tissue fixation and an outer anti-adhesive surface to prevent irritation to adjacent structures, such as the tongue [[157], [158], [159], [160], [161], [162]]. Beyond asymmetry, researchers have drawn inspiration from nature to fabricate biomimetic microstructures that mimic octopus suckers, beetle patterns, and Boston ivy [[163], [164], [165], [166], [167], [168]] to facilitate mechanical interlocking and localized negative pressure (Fig. 5A) [[172], [173], [174]]. A prominent example is the construction of hydrogels with a twisted plywood structure, mimicking the crustacean exoskeleton. By integrating liquid crystal self-assembly with nanocrystal engineering, these systemic architectures have achieved an unprecedented tensile strength of 46 MPa and a fatigue threshold of 32.5 kJ per square meter [175].

To translate these sophisticated bio-inspired designs into clinically viable products, scalable manufacturing technologies are being integrated into the design framework. High-throughput strategies, such as UV photolithography, now enable the creation of complex 3D shape-changing hydrogels by programming anisotropic microstructures and internal stress fields. By utilizing different photomasks to regulate local gradient structures, hydrogels can be mass-produced into intricate configurations, such as bionic avian or fighter-jet shapes, which exhibit novel 3D-to-3D transformation modes [176]. These manufacturing advancements, along with automated template-assisted micro-molding, ensure structural fidelity and functional reliability across large-scale batches, bridging the gap between delicate biomimetic engineering and industrial production.

The second dimension involves dynamic responsiveness, where the system actively adapts to environmental cues through external or internal triggers. External adaptation strategies utilize physical energy fields to drive molecular and network-level events as needed. Recent cross-hierarchical innovations have focused on creating feedback loops between sensing and action at different scales. For instance, ultrasound-mediated cavitation actively embeds molecular sutures into biological tissues, whereas near-infrared light-triggered photothermal materials drive the penetration of conductive polymers, thereby establishing seamless electrical interfaces [130].

Complementing these external fields, internal stimuli-responsive designs enable the system to perceive and react to pathological signals [[177], [178], [179], [180], [181], [182]]. For example (Fig. 5B), incorporating pH-sensitive chemical bonds, such as boronate esters, enhances the adhesive strength of the gel in the acidic environment of periodontitis or ulcers [169,[183], [184], [185]]. Utilizing MMP-sensitive peptides as crosslinkers facilitates the specific degradation of the gel by highly expressed matrix metalloproteinases at inflammatory sites, triggering targeted drug release [170,[186], [187], [188]]. Furthermore, the development of semiconvertible supramolecular hydrogels facilitates controllable transitions between adhesive and non-adhesive states, enabling on-demand detachment without tissue trauma [171,[189], [190], [191]]. This hierarchical coordination, where molecular-level sensing drives system-level execution, ensures that the adhesive system acts as an integrated living device capable of personalized and precise therapy. In essence, system-level integration exemplifies the highest form of synergistic design, where molecular sensing, network reconfiguration, and macroscopic function are computationally orchestrated to create adaptive, life-like therapeutic interfaces.

### 4.4 Performance validation: from design blueprint to experimental confirmation

Beyond the multi-scale design of the hydrogels themselves establishing robust performance validation protocols is critical for clinical translation. The current assessment framework relies on multidimensional indicators as summarized in Table 2. Adhesive performance is quantitatively evaluated through standardized mechanical tests including tensile, lap shear, and peel tests to assess interfacial strength under various loading conditions [192]. Traditional in vitro assays often utilize biomimetic substrates such as porcine buccal mucosa or enamel slices [196]. To ensure structural integrity against the intricate physiological loading caused by chewing the bulk mechanical properties of the hydrogel are further characterized through compression, indentation, and rheological analyses [193,[197], [198], [199]]. Furthermore, environmental adaptability is assessed via specialized protocols such as cyclic peeling for fatigue resistance and underwater tests for stability in wet conditions [194,195,200,201]. Current methodologies often compartmentalize evaluation into isolated parameters, while these tests provide fundamental data, they collectively fail to recreate the integrated and dynamic pathophysiology of the oral cavity, where salivary clearance, masticatory stress, and microbial activity act concurrently to challenge material integrity.

**Table 2:** Key performance evaluation methods for adhesive hydrogels.

Evaluation Category	Test Methods	Key Performance Indicators	Representative Methods & Models	Refs.
Adhesive Performance	• Tensile adhesion tests • Lap shear tests • Peel tests	• Adhesive strength • Shear adhesion strength • Peel strength		[192]
Mechanical Performance	• Compression tests • Uniaxial tensile tests • Indentation tests	• Compression tests • Tensile strength • Elastic modulus		[193]
Environmental Adaptability & Durability	• Cyclic peeling tests • Underwater adhesion tests • Rheological tests	• Fatigue resistance • Adhesion durability in wet conditions • Self-healing ability		[194,195]

The fundamental discrepancy between simplified in vitro models and complex in vivo performance has long remained a persistent hurdle in bioadhesive research. Pivotal early work by Needleman and Smales underscored the inadequacy of static detachment force measurements by introducing an organ culture model that integrated dynamic fluid flow [202]. Their findings elucidated that adhesive duration, which represents a paramount clinical metric, is governed by polymer swelling and mucus interactions under dynamic conditions. These variables are fundamentally neglected in conventional tensile testing. Advancing this paradigm, Madsen et al. refined the conceptual framework of dynamic assessment by systematically investigating the influence of the test medium [203]. They demonstrated that the physicochemical properties of the irrigation fluid are decisive, establishing that only media closely replicating human saliva, especially regarding mucin content and rheological behavior, can yield bioadhesive retention profiles congruent with the in vivo state. This body of work emphasized that predictive validation must encapsulate the bifurcated bioadhesion process consisting of initial contact and a subsequent consolidation phase driven by hydration and intermolecular entanglement. The quest for a truly predictive methodology culminated in a comprehensive evaluation by Baus et al., who benchmarked various characterization techniques against actual human mucosal residence times [35]. Their study established that optimized ex vivo flow-retention tests under artificial saliva perfusion achieve an exceptional correlation with in vivo data (r = 0.973). Such evidence solidifies the superiority of dynamic evaluation under standardized washout conditions over traditional tensile studies for forecasting clinical outcomes.

Nevertheless, even these sophisticated dynamic models often treat mechanical fatigue and microbial challenges as independent variables, thereby failing to capture their synergistic impact. To bridge the remaining gap toward clinical reality, future standardization must prioritize the development of integrated bioreactor platforms. These next-generation systems should be engineered to simultaneously exert cyclic mechanical loading and dynamic salivary flow while exposing materials to complex multispecies oral biofilms. Such a holistic approach is imperative to elucidate the bio-mechanical crosstalk where microbial enzymatic activity may accelerate the fatigue failure of the adhesive interface. Transitioning from isolated parameter analysis toward this integrated validation paradigm will be indispensable for accurately predicting the clinical durability of next-generation oral hydrogels. This evolution ensures that materials are designed to maintain their functional integrity amidst the multifaceted and simultaneous stressors inherent to the human oral environment.

## 5 Applications of adhesive hydrogels in oral diseases

Adhesive hydrogels are being explored for the treatment of diverse oral diseases owing to their unique ability to maintain stable adhesion in moist environments and enable controlled drug release, offering therapeutic advantages. The current research landscape primarily focuses on major oral pathologies, including periodontal disease, dental caries, mucosal ulcers, infectious conditions, and precancerous lesions.

### 5.1 Application of Adhesive Hydrogels in Periodontitis Treatment

**Fig. 6:**
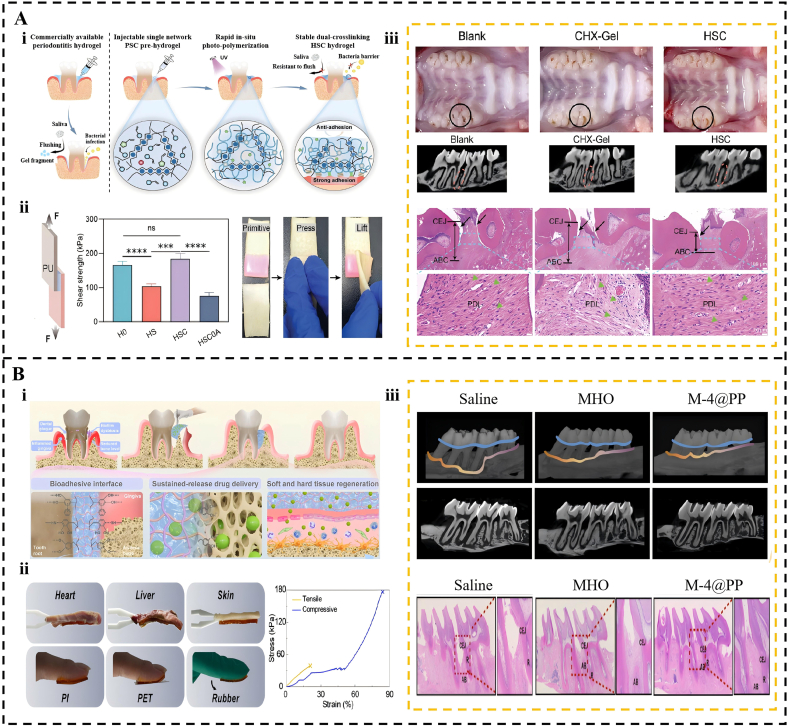
Application of Adhesive Hydrogels in Periodontitis Treatment. (A) A light-curable alginate-calcium hydrogel with enhanced wet adhesion for periodontal treatment, effectively blocking bacterial invasion. (i) Schematic diagram of in-situ photopolymerized hydrogel for periodontal disease treatment. (ii) Adhesion properties of hydrogels. (iii) In vivo periodontitis treatment. Reproduced with permission from Ref. [207]. Copyright 2024 John Wiley and Sons. (B) A bioinspired dual-network hydrogel for synergistic antibacterial and regenerative therapy of periodontitis. (i) Schematic illustration of the M@PP hydrogel for periodontitis treatment. (ii) The adhesion performance of M@PP hydrogel. (iii) Micro-CT scan images of maxillary alveolar bone in each group of rats and Histological staining analysis. Reproduced with permission from Ref. [208]. Copyright 2025 Elsevier.

Therapeutic efficacy in periodontitis is significantly compromised by the unique microenvironment of deep periodontal pockets, where persistent inflammation, complex pathogenic biofilms, and mechanical stresses from salivary flow and mastication collectively hinder effective drug delivery. In this context, adhesive hydrogels have emerged as promising solutions due to their injectability and superior wettable surface adhesion, enabling targeted intervention and prolonged retention at the disease site [[204], [205], [206]]. Initial research prioritized foundational adhesivity and injectability to ensure stable interfacial contact at the disease site. The in situ photocured HSC hydrogel represents a significant advancement, ensuring robust tissue integration through a tough double-network structure [207]. This system effectively overcomes the retention limitations of conventional topical therapies. Commercial chlorhexidine gel (CHX-Gel) typically disappears within 0.5 h owing to rapid salivary clearance. In contrast, the HSC system maintains 100% mucosal retention over 120 h while providing a high gingival adhesion strength of 275.62 kPa. In vivo evaluations have further validated these functional benefits. The HSC hydrogel significantly reduced the CEJ-ABC distance to 600 μm compared to 900 μm in the blank control group, as shown in Fig. 6A. These quantitative improvements confirm the superior capacity of this adhesive platform to enhance bone preservation and minimize tissue absorption. However, this system typically exhibits passive drug release profiles that do not align with the dynamic pathology of periodontitis. Research has shifted toward environmentally responsive, intelligent adhesive systems. Kim et al. developed an Alg-PBA/TA hydrogel that maintains stable adhesion and sustains antibacterial activity under fluctuating pH conditions through an ingenious dynamic covalent chemistry design [209]. The novel NHRT hydrogel further improves this by simultaneously sensing bacterial enzymes and inflammatory signals to trigger distinct therapeutic programs [210].

The evolution of drug delivery platforms further addresses the specific clinical shortcomings of conventional antibiotics. Standard therapies, such as 2% minocycline ointment, demonstrate initial efficacy by reducing probing depth and bleeding on probing [211]. However, their clinical utility is restricted by rapid clearance within 24 h. These ointments also exhibit an initial cytotoxic burst release exceeding 1300 μg/mL. The M@PP hydrogel platform overcomes these critical safety and retention issues by maintaining therapeutic concentrations below 15 μg/mL for over 60 h [208]. This material possesses a robust mechanical strength of 177.2 kPa. It also promotes a 143.37% increase in the bone volume fraction through targeted immunomodulation, as illustrated in Fig. 6B. Specialized platforms also target the metabolic challenges of diabetic periodontitis by synergizing antioxidant microfibers with the targeted release of metformin or hydrogen sulfide to effectively reverse hyperglycemic inflammation [[204], [205], [206]]. Through this controlled release mechanism, the M@PP system achieves superior regenerative outcomes while minimizing local tissue damage. Building on these sustained delivery strategies, research has progressed toward sequential delivery to better align with the natural stages of tissue healing. For example, the MB/Sema3A@SA-DA hydrogel coordinates an initial 14-h antimicrobial burst with a 28-day regenerative program [212]. This temporal control more effectively facilitates bone healing by providing a 71.96% increase in the bone volume fraction compared to single-factor formulations. The TOOTH hydrogel also utilizes staged release strategies to orchestrate microenvironmental conditioning [213]. The functional scope of these materials has been extended to integrated bioactivation. Innovations include piezoelectric hydrogels that transduce masticatory forces into osteogenic electrical stimuli [214,215]. Notably, the development of F127DA hydrogels replicates the biomechanics of native tissues to activate mechanotransduction pathways in stem cells [216]. These developments signify a paradigm shift from passive drug carriers to active scaffolds.

Despite these promising laboratory results, clinical translation from rodent models to human patients remains challenging. The transition is complicated by significant anatomical discrepancies because human periodontal pockets are far deeper and more irregular than shallow rat sockets [217]. Furthermore, the aggressive polymicrobial biofilms in the human oral cavity accelerate material degradation beyond that observed in controlled animal inoculations [218,219]. Therefore, the principal challenge is not merely extending adhesion duration but engineering hydrogels with rheological properties that ensure conformal filling of complex pocket anatomy coupled with sustained anti-biofilm and immunomodulatory functions over the 3 to 4 week period required for human periodontal tissue regeneration.

### 5.2 Application of Adhesive Hydrogels in Dental Caries Management

**Fig. 7:**
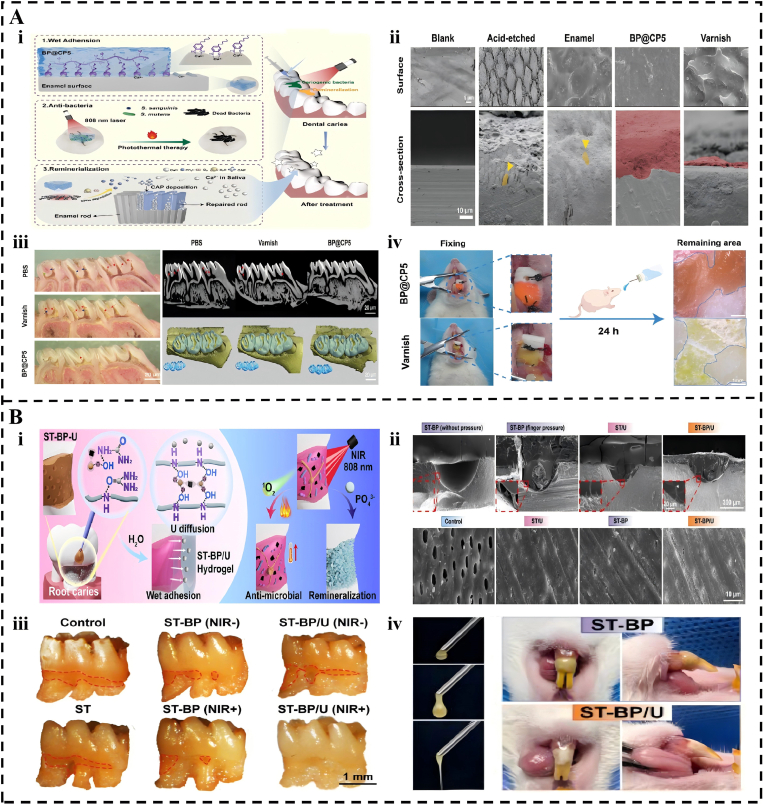
Application of Adhesive Hydrogels in Dental Caries Management. (A) A phototherapy adhesive hydrogel for antibacterial and enamel repair in early caries. (i) Schematic illustration of the preparation of the BP@CP5 hydrogel. (ii) The corresponding cross-sectional SEM images after various treatments. (iii) In Vivo Dental Caries Prevention Effectiveness of BP@CP5. (iv) Adhesive properties of BP@CP were compared with the blank control group in vivo. Reproduced with permission from Ref. [221]. Copyright John Wiley and Sons. (B) A hagfish-inspired fluid-to-gel system for photothermal antibacterial therapy and dentin remineralization in root caries treatment. (i) The application of ST-BP-U to root caries. (ii) ST-BP-U spreads on the tooth surface and penetrates the dentinal tubules. (iii) Caries staining shows differences in rat first molars across groups. (iv) ST-BP-U injection and ST and ST-BP/U attached to the tooth surface. Reproduced with permission from Ref. [222]. Copyright 2024 Elsevier.

Dental caries result from the demineralization of tooth enamel due to acid production by cariogenic bacteria such as *Streptococcus mutans* [220]. Consequently, adhesive hydrogels are essential for the prevention and treatment of caries, as they resist salivary washout and ensure long-term retention and controlled release of functional agents on the tooth surface, thus inhibiting cariogenic biofilms and facilitating tooth tissue remineralization. Adhesive hydrogels demonstrate superior clinical potential compared to traditional fluoride varnishes and topical antibiotics. The BP@CP5 system achieves a lap shear strength of 24.3 kPa on tooth enamel [221]. This value is significantly higher than the 3.3 kPa recorded for the standard fluoride varnish. In vivo evaluations have revealed that BP@CP5 maintains a 53.5% retention rate after 24 h of active masticatory activity (Fig. 7A). It achieves a bacterial killing rate exceeding 97%, whereas traditional varnishes reduce bacterial populations by only 19%. Furthermore, the resulting mineral layer reaches a hardness of 255.91 GPa. This value is significantly closer to that of healthy enamel than the 201.19 GPa achieved by conventional fluoride treatments. This platform effectively avoids clinical adverse effects, such as dental fluorosis and tooth staining associated with chlorhexidine.

Advanced designs overcome the penetration limitations of traditional agents for challenging lesions, such as root caries. Specifically, for irregular root surface caries, the STAG hydrogel designed by Li et al. exhibits excellent flowability and in situ gelling properties, enabling the complete filling of carious cavities and the formation of a stable barrier while promoting remineralization through the release of amorphous calcium phosphate (ACP) [223]. Similarly, advanced systems show marked improvements over conventional topical agents that struggle with biofilm penetration and retention. The ST-BP-U system addresses the challenges posed by irregular root surface caries through its superior penetration capabilities [222]. This hagfish-inspired hydrogel penetrates dentinal tubules to a depth of 167.2 μm, far exceeding the 50 μm limit of standard silk fibroin gels. It exhibits a robust wet adhesion strength of 270.3 kPa and eliminates 92.6% of mature biofilms. This performance represents a major improvement over conventional topical agents, which struggle with biofilm resistance. In rat models, this treatment increases mineral density to 1476.6 mg/cm^3^ and successfully prevents further demineralization (Fig. 7B). By providing a stable micro-mechanical interlock, the ST-BP-U system maintains structural integrity in moist environments, where commercial resins often fail.

To mitigate pulp damage caused by deep caries, regenerative endodontic treatment (RET) requires support for the viability and differentiation of dental pulp stem cells [224]. Moving beyond the merely antimicrobial or sealing functions of traditional materials, the chitosan–alginate composite hydrogel (C/A hydrogel), loaded with an extracellular matrix (ECM) derived from pulp and dentin, provides a highly biomimetic microenvironment for human dental pulp stem cells (hDPSCs). It significantly enhances their differentiation into odontoblast-like cells, demonstrating substantial potential for pulp regeneration [225]. When combined with growth factor delivery systems, such materials are expected to advance regenerative endodontics toward clinical application and offer a biological solution for the repair of pulp injuries [226,227].

The design of adhesive hydrogels for caries management faces distinct challenges that are specific to the therapeutic target. For enamel and dentin repair the primary objective is to replicate the complex prismatic crystal structure of natural tooth tissue. Current strategies often fall short in achieving organized biomimetic mineralization. A more fundamental limitation lies in ensuring a durable marginal seal that can withstand constant masticatory fatigue over months to prevent secondary caries, a duration far exceeding the 7 to 14 days of interfacial integrity demonstrated by most experimental hydrogels. For regenerative endodontic therapies the limitations shift from structural to biological, centering on achieving precise spatiotemporal control over growth factor release and engineering functional vascularization within the confined root canal space.

### 5.3 Application of Adhesive Hydrogels in Oral Ulcers

Oral ulcers are a common type of oral mucosal lesion, frequently accompanied by pain, inflammation, and impaired wound healing, which significantly impact patient quality of life. The therapeutic demands of oral ulcers are driving the evolution of adhesive hydrogels from passive dressings to active healing platforms. The primary objective is to achieve stable adhesion on exudate-covered wound surfaces while preventing secondary damage to surrounding healthy tissues [228]. The Janus patch developed by Zhang et al. exemplifies this approach, with its adhesive side securing to the wound through strong chemical interactions, while its lubricious side reduces friction with healthy mucosa, addressing the balance between secure adhesion and preventing additional injury [229].

Based on stable coverage, the pursuit of multifunctional synergistic therapies to actively accelerate healing has become a primary focus of contemporary research. For example, Zhu et al. developed a GNT dual-crosslinked hydrogel incorporating tannic acid, which offers multiple bioactive functionalities, including rapid hemostasis, potent anti-inflammatory effects by inhibiting pro-inflammatory cytokines such as TNF-*α* and IL-17, and promotion of epithelial and vascular regeneration [230]. Similarly, Wang et al. developed a CHG/GelMA/M-HA hydrogel that combines catechol-mediated wet adhesion, sustained chlorhexidine (CHG) release for antimicrobial effectiveness, and the immunomodulatory properties of modified hyaluronic acid (M-HA), thereby achieving triple therapeutic synergy [231].

**Fig. 8:**
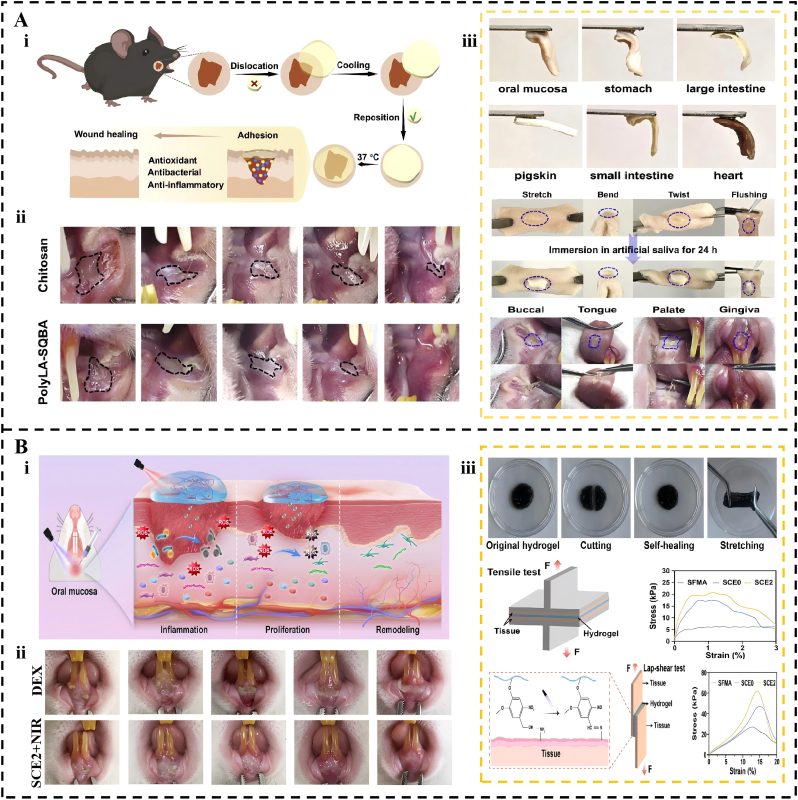
Application of Adhesive Hydrogels in Oral Ulcers. (A) A thermosensitive, self-healing adhesive with antibacterial and antioxidant properties for accelerated oral ulcer healing. (i) Schematic diagram of application of the PolyLA-SQBA hydrogel. (ii) Photographs of rat oral ulcers treated by PolyLA-SQBA hydrogel and chitosan patch at 0, 2, 4, 6, and 8 days. (iii) Adhesion characteristics of PolyLA-SQBA hydrogels. Reproduced with permission from Ref. [235]. Copyright 2025 Elsevier. (B) A UV-adhesive hydrogel patch with photothermal antibacterial and antioxidant activities for accelerated healing of oral ulcers, including in diabetic conditions. (i) The SCE2 hydrogel was developed and employed to expedite the healing process of oral ulcers. (ii) Comparison of SCE2 hydrogel versus dexamethasone (DEX) in promoting healing of MRSA-infected diabetic oral ulcers in vivo. (iii) The self-healing capabilities and adhesive strength of SCE2 hydrogel. Reproduced with permission from Ref. [236]. Copyright 2024 Elsevier.

Systems capable of intelligent responses represent a cutting-edge approach that directly addresses the shortcomings of traditional clinical treatments. These advanced systems are designed to sense and dynamically adapt to complex pathological microenvironments [[232], [233], [234]]. For instance, the thermoresponsive PolyLA-SQBA hydrogel offers a substantial improvement over commercial chitosan patches, maintaining stable mucosal adhesion for approximately 24 h, whereas standard patches often dissolve within only 3 h [235]. Correspondingly, in rat ulcer models, the PolyLA-SQBA system achieves a healing rate of 93.03% by the eighth day, significantly surpassing the 78.4% recorded for the commercial control (Fig. 8A). Another sophisticated approach is the light-controlled SCE2 hydrogel, which addresses the shortcomings of conventional glucocorticoid solutions, such as dexamethasone (DEX) [236]. The SCE2 system achieves a robust peak shear strength of 64.37 kPa through light-triggered Schiff base reactions (Fig. 8B). In diabetic oral ulcer models, the SCE2 platform, when combined with near-infrared irradiation, leads to nearly complete healing by the fifth day. In contrast, the dexamethasone group often exhibits persistent epidermal necrosis and significant inflammatory cell infiltration during the same period. The functional scope of these materials continues to expand toward treating refractory wounds, such as diabetic oral ulcers. The MPG3 biomimetic hydrogel effectively scavenges reactive oxygen species and modulates macrophage phenotypes to promote healing [237].

The treatment of oral ulcers requires a multifunctional adhesive dressing that provides a stable physical barrier while actively promoting healing. The core challenge extends beyond adhesion duration to managing the dynamic ulcer microenvironment. Successful hydrogels must simultaneously fulfill conflicting demands rapid yet gentle adhesion on wet, sensitive tissue effective management of inflammatory exudate without swelling or delamination, and maintaining mechanical compliance to withstand constant tongue and speech movement without causing discomfort or secondary trauma. Future designs must therefore integrate smart fluid-handling capabilities with tunable viscoelasticity, ensuring patient comfort alongside therapeutic efficacy throughout the healing process.

### 5.4 Adhesive hydrogels for the Treatment of Oral Fungal and Viral Infections

**Fig. 9:**
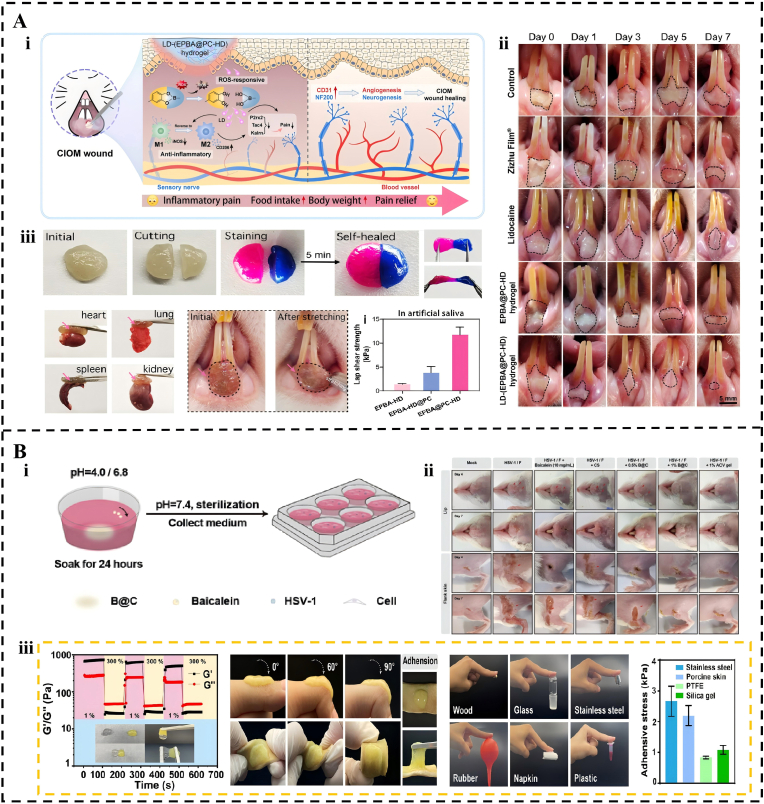
Adhesive Hydrogels for the Treatment of Oral Fungal and Viral Infections. (A) Surface-engineered hydrophobic hydrogel via cholesterol micelle rearrangement. (i) Wound repair mechanism of the EPBA@PC-HD hydrogel in CIOM rats. (ii) Treatment of CIOM wounds with EPBA@PC-HD hydrogel versus conventional therapies over 7 days. (iii) Macroscopic self-healing and adhesion of the EPBA@PC-HD hydrogel. Reproduced with permission from Ref. [239]. Copyright 2025 Elsevier. (B) A pH-responsive baicalein-chitosan hydrogel for targeted therapy against HSV-1 skin infections, including drug-resistant strains. (i) Schematic of the in vitro assay assessing the anti-HSV-1 activity of B@C hydrogels. (ii) Efficacy against HSV-1 infection in the murine labial model and flank skin. (iii) Self-healing capability, skin spreadability, and adhesion performance of the 1% B@C hydrogel. Reproduced with permission from Ref. [240]. Copyright 2025 John Wiley and Sons.

The pathogenesis of oral infections has a multifactorial etiology, involving bacterial, fungal, and viral pathogens. Adhesive hydrogels provide an effective platform for prolonging drug action and increasing local concentration, particularly in managing complex conditions where inflammation and infection coexist, such as chemotherapy-induced oral mucositis (CIOM) [238]. For instance, the EPBA@PC-HD hydrogel addresses the rigorous requirements of such environments by employing a micelle rearrangement strategy to repel interfacial water [239]. This system achieves a wet adhesion strength of approximately 12 kPa, which is six times that of standard polyelectrolyte networks. In vivo results have demonstrated that the lidocaine-loaded version achieves a healing rate of 93.60% within seven days (Fig. 9A). This performance significantly exceeds that of commercial products, such as Zizhu Propolis Film, and effectively restores food intake by alleviating inflammatory pain. Similarly, to address patient compliance issues related to pain, Kan et al. [241] employed a deep dopamine modification strategy to create a hydrogel with ultra-strong wet adhesion, ensuring reliable retention even on painful and inflamed mucosa. Building upon reliable adhesion, research has advanced to incorporate active anti-inflammatory and antimicrobial functionalities [[241], [242], [243]]. Shao et al. [244] integrated the natural anti-inflammatory and pro-adhesive compound EGCG into a thermosensitive hydrogel, achieving a synergy where in situ gelation enhances localized drug delivery. Additionally, Pan et al. [245] developed a semi-interpenetrating network hydrogel loaded with both oridonin (ORI) and DNase I. This dual-drug system enables the controlled release of ORI to suppress inflammation, while DNase I degrades neutrophil extracellular traps (NETs), effectively interrupting a key amplification loop of infection-associated inflammation.

Adhesive hydrogels also exhibit high adaptability to diverse fungal and perioral viral infections. For oral candidiasis thermosensitive gels incorporating natural red ginger extract or peppermint oil significantly prolong the retention of antifungal components to effectively inhibit *Candida albicans*. Research by Kam et al. [246] demonstrated that optimizing carbomer concentrations in red ginger formulations can simultaneously enhance adhesion and patient comfort. Similarly, Prakriti et al. [247] utilized a Carbopol 940-based network to sustain the delivery of peppermint essential oil at the infection site. For the treatment of viral infections, such as herpes labialis caused by HSV-1, Redín et al. [248] introduced a 5% acyclovir bioadhesive gel that achieved considerably higher drug retention in the skin compared to commercial formulations by optimizing its film-forming and adhesive properties, thereby enhancing local antiviral efficacy. The pH-responsive B@C hydrogel addresses the shortcomings of conventional antiviral agents against HSV-1 [240]. While Acyclovir (ACV) remains a clinical benchmark, its efficacy is often hindered by drug-resistant strains. The B@C system overcomes this challenge by ensuring sustained retention of Baicalin for over 72 h and achieving a 75% reduction in viral latency in perioral infection models (Fig. 9B). This platform also demonstrates high efficacy against ACV-resistant HSV-1 strains, reducing the infection rate to 2% under acidic conditions. Targeted application leads to scarless healing by the seventh day, which represents a significant improvement over free drug formulations.

Although adhesive hydrogels effectively extend drug duration, several challenges hinder their clinical translation for infection management. The wet adhesion strength and functional integrity of current systems, while improved, are often outmatched by the persistent and recurrent nature of oral infections which demand sustained local protection over days to weeks. More critically, the non-selective nature of many broad-spectrum antimicrobial hydrogels risks disrupting the ecological balance of the commensal oral microbiome, potentially leading to dysbiosis or secondary infections. Future research must therefore pivot from indiscriminate antimicrobial action toward precision strategies. This includes developing intelligent hydrogels capable of specific pathogen targeting and innovating ecological therapies aimed at restoring and maintaining microbial homeostasis, thereby addressing both the infection and the integrity of the oral ecosystem.

### 5.5 Applications of Adhesive Hydrogels in Precancerous Lesions

**Fig. 10:**
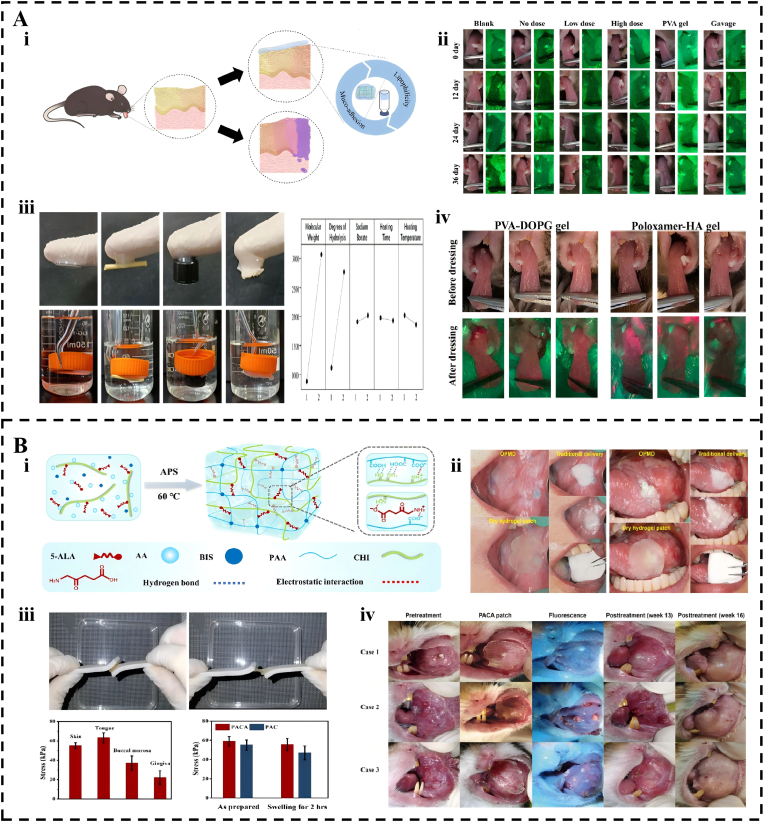
Applications of Adhesive Hydrogels in Precancerous Lesions. (A) A dual-adhesive/lipophilic PVA-DOPG hydrogel delivering rapamycin to inhibit oral leukoplakia malignant transformation. (i) Schematic summary of hydrogel preventing malignant transformation of OLK. (ii) Preventive efficacy validated by visual and VEL scope imaging of the 4-NQO-induced OLK model. (iii) Adhesion of PVA-DOPG Rap hydrogel in air versus water across diverse substrates (glass, wood, plastic, porcine mucosa). (iv) Red fluorescence of hypericin detected by VEL scope, showing its penetration through the keratinized mucosa. Reproduced with permission from Ref. [249]. Copyright 2024 Elsevier. (B) A strongly mucosal-adhesive PAA-chitosan hydrogel patch for enhanced ALA delivery in patient-friendly photodynamic therapy of oral potentially malignant disorders. (i) Schematic diagram of the synthesis of PACA patch. (ii) Comfort trial of the PAA-CHI hydrogel patch in participants with OPMDs. (iii) The adhesion strength of PACA hydrogel patch. (iv) PACA-PDT treatment of DMBA-induced buccal pouch carcinogenesis in hamsters. Reproduced with permission from Ref. [250]. Copyright 2022 Elsevier.

Clinical interventions targeting oral potentially malignant disorders (OPMDs), such as leukoplakia, aim to prevent their transformation into malignant tumors. Traditional surgical excision remains a standard approach; however, it is often associated with functional impairment and high recurrence rates ranging from 30% to 50%. Adhesive hydrogels offer a versatile and minimally invasive platform for overcoming these limitations by ensuring sustained drug delivery. For instance, Du et al. [249] developed an adhesive PVA-DOPG hydrogel that significantly enhances the penetration of molecules, such as rapamycin, through a keratinized mucosal layer. This system achieves an average penetration depth of 0.96 mm, which markedly outperforms conventional hydrophilic hydrogels. In mouse models of oral leukoplakia, the group receiving high-dose treatment showed a 36.36% reduction in tumor incidence density and achieved a volume inhibition rate of 75.04% while significantly improving animal survival. This quantitative evidence demonstrates that combining strong adhesion with high lipophilicity can overcome the delivery barriers that hinder traditional topical drugs (Fig. 10A).

Current advanced strategies primarily focus on localized and targeted therapies. Hydrogel-based local immunotherapy effectively activates the antitumor immune response by gradually releasing immune checkpoint inhibitors (e.g., anti-PD-1 antibodies) or innate immune agonists [251,252]. Adhesive hydrogels further enhance the efficacy of traditional therapies. For instance, Yu et al. developed a chitosan/fucoidan composite hydrogel loaded with the corticosteroid triamcinolone acetonide. This adhesive patch is engineered to enhance mechanical integrity and mucosal adhesion, providing a sustained release of the immunomodulatory drug to suppress the local inflammatory microenvironment in OPMDs [253]. Photodynamic therapy (PDT) effectively treats OPMDs but is limited by the stability and penetration of the photosensitizer [254]. Conventional ALA-PDT protocols typically utilize 20% ALA solutions supported by cumbersome gauze and starch films, which are easily displaced by saliva and tongue movements. Wang et al. [250] developed an interpenetrating network hydrogel (PACA) that leverages superior wet adhesion to achieve sustained and stable release of the photosensitizer ALA at the lesion site (Fig. 10B). This dry-crosslinking platform achieves an adhesion strength of 62 kPa within 10 s, effectively inhibiting mucosal dysplasia. Notably, in DMBA-induced hamster models, PACA-mediated PDT showed superior efficacy compared to traditional ALA solution-based PDT. This resulted in a significant reduction in tumor volume to 3.01 ± 0.92 mm^3^ at week 14 compared to 21.19 ± 4.52 mm^3^ in the liquid ALA group. This performance enabled complete lesion resolution approximately two weeks earlier than that achieved using standard protocols. Furthermore, a pilot patient survey has indicated significantly higher satisfaction with the hydrogel patch, which received an average score of 4.83 compared to only 1.66 for traditional ALA application. This increased satisfaction is due to the minimized interference with speech and swallowing. Beyond PDT, molecular-targeted therapies have emerged, such as supramolecular adhesive hydrogels utilizing boronate ester bonds to target dysplastic keratinocytes through the controlled release of isoguanosine [255].

Addressing oral precancerous lesions presents unique clinical hurdles beyond simple adhesion. The fundamental challenge lies in achieving efficient drug delivery across the protective mucosal barrier to reach the basal layer where dysplastic cells reside. While current hydrogels improve surface retention, their limited adhesion duration in the dynamic oral environment often fails to support the prolonged drug exposure needed for effective chemoprevention. Moreover, therapeutic success critically depends on patient compliance with long-term topical regimens, necessitating designs that are not only effective but also convenient and comfortable for daily use over weeks or months. Future innovations must therefore focus on hydrogels with enhanced transmucosal penetration capabilities and user-centric designs that integrate seamlessly into patient routines, thereby addressing both the biological and behavioral dimensions of oral cancer prevention.

### 5.6 Applications of adhesive hydrogels in oral and maxillofacial surgery

**Fig. 11:**
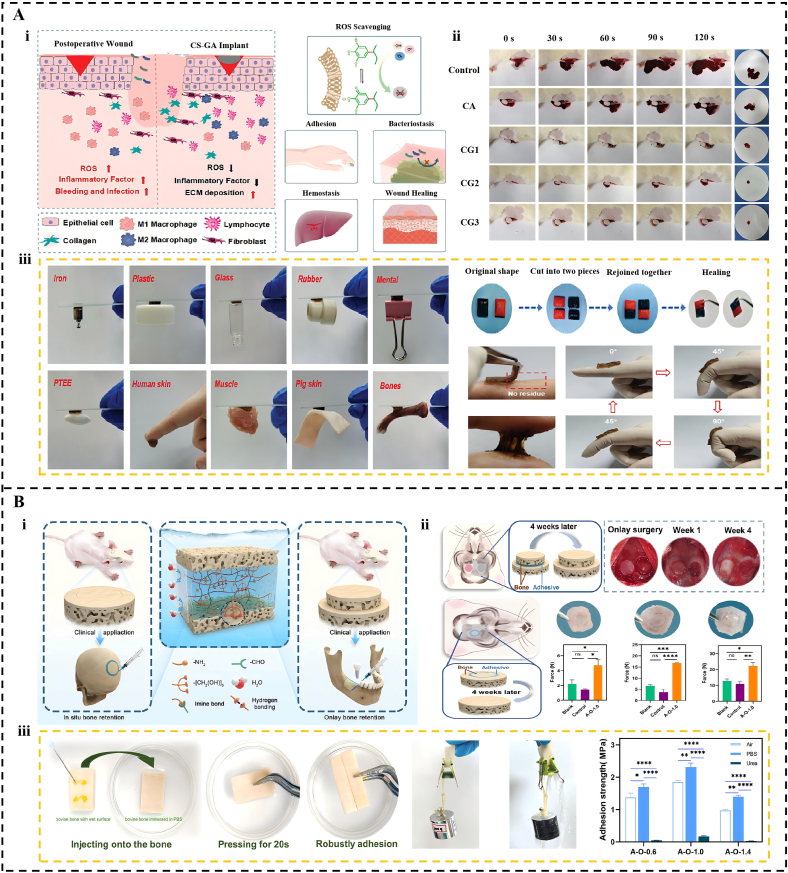
Application of Adhesive Hydrogels in Oral Ulcers. (A) A multifunctional chitosan-gallic acid hydrogel dressing with strong adhesion, antibacterial, antioxidant, and pro-healing properties for postoperative wound care. (i) Schematic diagram showing the synthesis of CS-GA for potential application in periodontal tissue regeneration. (ii) Comparative hemostatic effect of CS-GA and other treatments. (iii) Adhesion, dynamic strip ability, and self-healing of CS-GA hydrogel. Reproduced with permission from Ref. [259]. Copyright 2024 John Wiley and Sons. (B) An injectable, double-crosslinked hydrogel with long-term underwater bone adhesion for maxillofacial bone fixation. (i) Design of the Double-Cross-Linked Hydrogel. (ii) In situ bone retention ability and onlay bone retention ability of the Hydrogel. (iii) In vitro bone adhesion capability of Hydrogels. Reproduced with permission from Ref. [260]. Copyright 2023 American Chemical Society.

Oral and maxillofacial surgery entails the repair of soft tissue wounds and the reconstruction of bone, necessitating materials with excellent biocompatibility, hemostatic capabilities, mechanical support, and effective integration with moist tissues. In soft tissue repair, adhesive hydrogels are optimal postoperative dressings [256,257]. Wu et al. [258] developed a biomimetic hydrogel dressing (EMH), inspired by the natural extracellular matrix, which achieved exceptional mechanical toughness and sustained wound protection through a controllable, low-swelling double-network structure. Building upon this advancement, Feng et al. [259] developed a chitosan-gallic acid (CS-GA) hydrogel that exhibits strong adhesivity, self-healing, and robust mechanical properties (Fig. 11A). This multifunctional adhesive achieves an adjustable lap shear strength of 41.8 kPa on moistened tissues and exhibits potent antioxidant activity through ROS scavenging. In rat palatal defect models, the CS-GA hydrogel effectively shortened the hemostasis window to under 30 s, whereas conventional gauze packing or suture techniques often require 2–3 min to achieve clot stabilization. Furthermore, the material accelerates tissue recovery by increasing collagen fiber deposition and upregulating vascular endothelial growth factor levels. By reducing the expression of pro-inflammatory markers, such as TNF-α, this platform maintains a stable microenvironment, even in the presence of oral pathogens such as *Streptococcus mutans*.

Beyond soft tissue repair, adhesive hydrogels offer distinct advantages for bone tissue reconstruction, where stable bone retention is key to successful grafting. Traditional invasive technologies, such as metal plates and screws, often cause complications such as stress shielding and foreign-body reactions, which may eventually necessitate a second operation [261,262]. In response to the requirements of maxillofacial bone preservation surgery, Yang et al. [260] developed a dual-crosslinked AO hydrogel to address the critical challenge of bone retention in wet oral-maxillofacial surgical environments (Fig. 11B). This hydrogel utilizes high-density hydroxyl clusters to achieve an exceptional underwater bone adhesion strength of 2.32 ± 0.21 MPa. This performance consistently surpasses that of commercial cyanoacrylate adhesives, such as Vetbond, by approximately 1.0 MPa. In situ bone retention models demonstrated that the A-O hydrogel group achieves a retention force of 22.28 N at four weeks. In contrast, commercial cyanoacrylate-based adhesives often lead to serious bone absorption owing to their rigid, dense interface, which blocks nutrient exchang. The biomimetic porous structure of the A-O hydrogel facilitates the migration of osteogenesis-related cells, thereby ensuring superior osseointegration compared to traditional PMMA or CA bone cements.

Building on stable mechanical retention, current research has advanced toward interactive scaffolds that actively modulate the bone-regenerative microenvironment. The nHA-modified Gel-ADA hydrogel utilizes a dual-crosslinking network to establish a highly ordered porous structure with pore diameters ranging from 10 to 30 μm [263]. This architecture perfectly matches the dimensions of bone marrow stromal cells, facilitating interactive communication and accelerating bone repair. Similarly, the MA-HA-Dopa hydrogel developed by Christoph et al. [264] serves as a minimally invasive delivery vehicle that enhances the survival of mesenchymal stem cells through a dopamine-based adhesive interface. These biomimetic scaffolds provide superior osteointegration compared to traditional PMMA or ceramic bone cements, which often lack the necessary porosity for effective cellular infiltration.

Significant challenges persist in achieving functional restoration of the intricate maxillofacial region where soft and hard tissues coexist. The principal limitation is no longer merely adhesion strength or duration, but the fundamental mechanical mismatch between homogeneous hydrogel scaffolds and the graded, composite nature of the oral tissue interface. A hydrogel must simultaneously conform to and adhere to resilient mucosa, robust gingiva, and rigid alveolar bone, all while withstanding complex, cyclic masticatory forces far exceeding those in dermal repair. Future progress hinges on engineering composite or gradient hydrogels that replicate this structural heterogeneity, enabling seamless biomechanical integration and directed regeneration of multiple tissue types within a single, stable construct.

## 6 Challenges and outlook for adhesive hydrogels in the treatment of oral diseases

Although adhesive hydrogels show considerable promise for localized oral therapy, the transition from laboratory designs to clinical products remains highly challenging. A fundamental translational obstacle arises from the considerable mechanical and physiological heterogeneity across different oral regions. The functional longevity of a hydrogel is not an intrinsic property but is determined by its interaction with the local microenvironment. For instance, the buccal mucosa primarily endures shear and compression from mastication, whereas the sublingual area is dominated by high-velocity salivary flow. Consequently, a formulation that adheres robustly in one region may undergo rapid detachment in another region. Furthermore, the irregular topographies of sites, such as the gingival margin and root caries lesions, impose additional degradation pathways. This lack of site-specific adaptation implies that most current designs fail to guarantee reliable performance across the diverse anatomical landscape of the oral cavity.

This gap is primarily due to the intricate physiology of the oral cavity and the rigorous requirements of translational pathways [265,266]. Clinical approval necessitates comprehensive evaluations of mucosal irritation and the potential systemic toxicity of degradation byproducts over prolonged periods. Current laboratory assessments often fail to replicate the complex interplay between materials and the diverse oral microbiota. This limitation renders the long-term biological safety of these scaffolds unpredictable under real-world conditions. Furthermore, a significant disparity exists between the sophisticated architectures of multifunctional hydrogels and their economic feasibility in routine dentistry. Advanced systems, such as Janus patches and multi-signal responsive networks, often rely on multistep chemical modifications and high-purity precursors [267,268]. These intricate designs lead to prohibitive production costs and substantial variability in the batch quality during large-scale manufacturing.

Such economic barriers hinder these materials from competing with established, lower-cost clinical alternatives [269,270]. To address these challenges, future research must prioritize the integration of simplified material design and cost-effective fabrication. Streamlining the synthesis process through modular assembly or utilizing biomass-derived precursors could significantly enhance scalability. Functionally, it is essential to create intelligent hydrogels that can accurately analyze complex microenvironments while remaining conducive to manufacturing. Ultimately, the transition to mainstream clinical use will depend on the establishment of a standardized performance evaluation system. This framework must move beyond universal testing to incorporate site-specific protocols that simulate the distinct hydrodynamic and biochemical challenges of target regions, such as the gingival sulcus.

Developing rigorous testing protocols for both mechanical durability and long-term biocompatibility remains a priority. Emerging techniques, such as 3D or 4D printing, offer promising pathways for creating tailored architectures with high precision and reduced waste [[271], [272], [273], [274]]. By aligning site-adapted design concepts with regulatory compliance and economic viability, researchers can bridge the current gap between experimental prototypes and reliable therapies. This comprehensive optimization, from basic synthesis to translational pathways, will be pivotal in determining the success of adhesive hydrogels in next-generation oral disease management.

## 7 Conclusion

In conclusion, adhesive hydrogels have emerged as a transformative class of materials for localized oral therapy owing to their exceptional interfacial adaptability and multifunctional potential. This review differentiates itself from previous studies by synthesizing research progress through a three-tier hierarchy that bridges molecular-level bonding with macroscopic mechanical resilience and system-level biological integration. Adhesive hydrogels effectively address the challenges of moist and high-shear oral environments by enabling precise drug delivery and extending the duration of action. The primary differentiator of this study lies in its focus on quantitative performance benchmarks and critical evaluation of biomechanical crosstalk, which dictates clinical success in addressing pathologies such as periodontitis and caries.

Despite these significant laboratory gains, the transition to clinical practice remains impeded by several fundamental bottlenecks, including the lack of site-specific adaptation and disparity between current adhesion times and human biological healing windows. The future success of this field depends on shifting from passive drug carriers toward intelligent and actively interacting scaffolds that can navigate the heterogeneous stressors of different oral regions. Furthermore, the most urgent requirement for the next generation of oral adhesives is the establishment of standardized and integrated evaluation models that simultaneously simulate dynamic fluidics, mechanical fatigue, and microbial challenges. By providing a specialized blueprint that aligns advanced design logic with regulatory and clinical realities, this study serves as a comprehensive guide for achieving stable and efficient therapy in next-generation oral medicine.

## CRediT authorship contribution statement

**Yufei Peng:** Writing – original draft. **Zhisheng Jiang:** Writing – original draft. **Shusen Xu:** Writing – review & editing. **Liming He:** Writing – review & editing. **Tong Jiang:** Writing – review & editing. **Yujie Yang:** Writing – review & editing. **Xiaoyan Xie:** Writing – review & editing. **Lanjie Lei:** Writing – review & editing.

## Declaration of competing interest

The authors declare the following financial interests/personal relationships which may be considered as potential competing interests: Xiaoyan Xie reports financial support was provided by Hunan Provincial Health Commission and Natural Science Foundation of Hunan Province. Zhisheng Jiang reports financial support was provided by Scientific Research Foundation of Hunan University of Chinese Medicine. If there are other authors, they declare that they have no known competing financial interests or personal relationships that could have appeared to influence the work reported in this paper.

## Data Availability

No data was used for the research described in the article.
